# Supplementary figures and images for: Differential transcriptome analysis and identification of genes related to resistance to blight in three varieties of Bambusa pervariabilis × Dendrocalamopsis grandis
Source: PeerJ. 2021 Oct 18;9:e12301. doi: 10.7717/peerj.12301 (PMC8530093; doi:10.7717/peerj.12301)

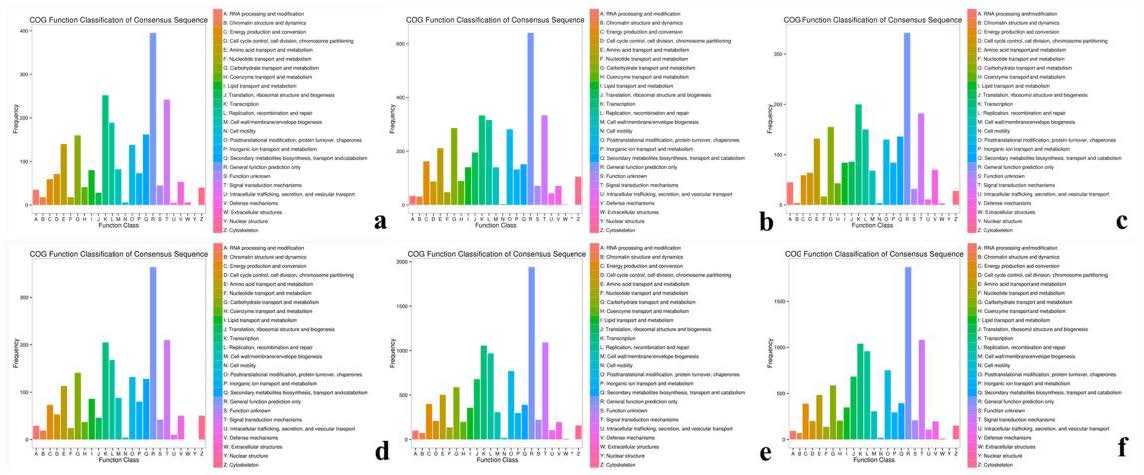

Supplement: Supplemental Information 4 — (A, B, C) COG classification diagram showing the DEGs between sterile water and spore suspension treatment at varieties #3, #6, 8 respectively; (D, E, F) COG classification diagram showing the DEGs between varieties #3 and #8, 6 and #8, #6 and #3 at spore suspension treatment respectively. [file peerj-09-12301-s004.jpg]

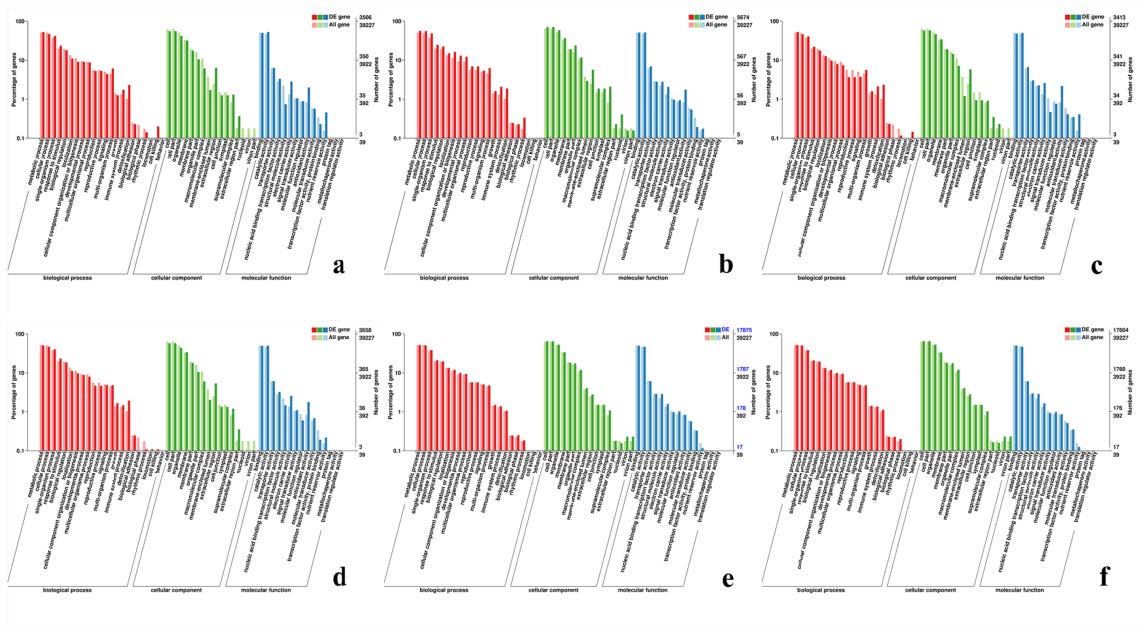

Supplement: Supplemental Information 5 — (A, B, C) GO classification diagram showing the DEGs between sterile water and spore suspension treatment at varieties #3, #6, #8 respectively; (D, E, F) GO classification diagram showing the DEGs between varieties #3 and #8, #6 and #8, #6 and #3 at spore suspension treatment respectively. [file peerj-09-12301-s005.jpg]

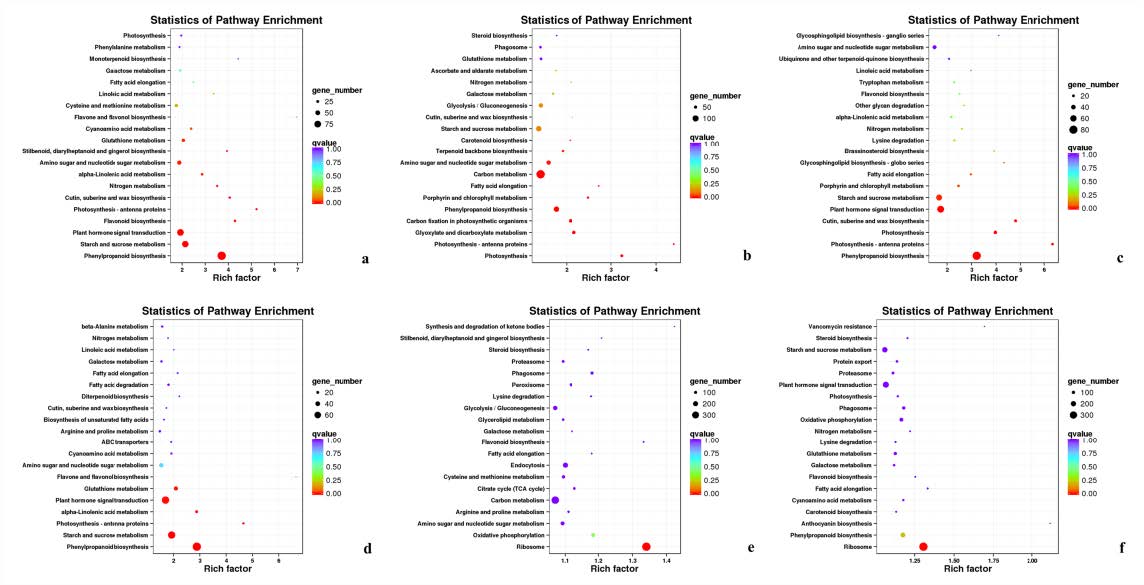

Supplement: Supplemental Information 6 — (A, B, C) KEGG classification diagram showing the DEGs between sterile water and spore suspension treatment at varieties #3, #6, #8 respectively; (D, E, F) KEGG classification diagram showing the DEGs between varieties #3 and #8, #6 and #8, #6 and #3 at spore suspension treatment respectively. [file peerj-09-12301-s006.jpg]
